# Genome-wide identification of circular RNAs in peanut (*Arachis hypogaea* L.)

**DOI:** 10.1186/s12864-019-6020-7

**Published:** 2019-08-15

**Authors:** Xingguo Zhang, Xingli Ma, Longlong Ning, Zhongfeng Li, Kunkun Zhao, Ke Li, Jialin He, Dongmei Yin

**Affiliations:** College of Agronomy, Henan Agricultural University, 95 Wenhua Road, Zhengzhou, Henan 450002 People’s Republic of China

**Keywords:** Peanut, Circular RNA, Genomic feature, Expression profile

## Abstract

**Background:**

Circular RNAs (circRNAs), a class of widely expressed endogenous regulatory RNAs, are involved in diverse physiological and developmental processes in eukaryotic cells. However, there have been no related studies on the number of circRNAs and their overall characteristics including circRNA abundance and expression profiles in peanut, which is one of the most important edible oil seed crops in the world.

**Results:**

We performed a genome-wide identification of circular RNAs using ribosomal-depleted RNA-sequencing from the seeds of two peanut eighth-generation recombinant inbred lines (RIL8): ‘RIL 8106’ (a medium-pod variety) and ‘RIL 8107’ (a super-pod variety), at 15 and 35 days after flowering (DAF), respectively. A total of 347 circRNA candidates were detected by two computational pipelines: CIRCexplorer and CIRI, with at least two supporting junction reads. All these circRNAs were generated from exons of annotated genes, and widespread on the 20 peanut chromosomes. The expression profiles revealed that circRNAs were differentially expressed between two stages and between two lines. GO enrichment analysis of the host genes produced differentially-expressed circRNAs suggested that circRNAs are involved in seed development and regulation of seed size. Fifteen circRNAs were experimentally analyzed by qRT-PCR with divergent primers, and six circRNAs were resistant to digestion with RNase R exonuclease, and the back-splicing sites were further validated by Sanger DNA sequencing.

**Conclusions:**

We present the first systematical investigation of the genomic characteristics and expression profiles of circRNAs in peanut. The results revealed that circRNAs are abundant and widespread in peanut, and the differentially-expressed circRNAs between two lines suggested that they might play regulatory roles in peanut seeds development.

**Electronic supplementary material:**

The online version of this article (10.1186/s12864-019-6020-7) contains supplementary material, which is available to authorized users.

## Background

Mature messenger RNAs (mRNAs) are linear molecules with clear termini that play important roles in molecular genetics. In addition to mRNA, cells also contain diverse types of non-coding RNA such as microRNAs (miRNA), small interfering RNAs (siRNA), long non-coding RNA (lncRNA) and circular RNAs (circRNAs). circRNAs, as a distinct class of newly discovered endogenous regulatory RNAs, were occasionally identified and had been considered to be splicing errors more than 20 years ago [1]. Recently, with the development of high-throughput RNA sequencing technology and highly efficient methods for analysis of big data sets, circRNAs have been identified widespread in animals and plants [2–6].

Circular RNA is covalently closed, single-stranded RNA molecule generated by back-splicing events. Recent studies have showed that circRNAs are not only abundantly and stably expressed in animal cells, but also play important roles in a wide range of biological and developmental processes in animals [2, 3, 7, 8]. The biogenesis of mammalian circRNAs is regulated by *cis*-regulatory elements such as repetitive sequences or non-repetitive reverse complementary sequences flanking the circularization exons, and *trans*-acting factors such as RNA binding protein [9–12]. An abundant circRNA in human cells, CDR1as, was observed to act as a “sponge” for multiple miRNAs and to regulate cell growth [3]. circRNAs have also been found to promote transcription of their parental coding genes [11, 13] and affecting the expression of parental genes by competitive generation with their linear counterparts [9, 14]. Although the functions of circRNAs are still largely unknown, their relative abundance, evolutionary conservation, and the fact that they are derived from important gene loci, means that circRNAs are the products of regulated back-splicing rather than by-products of splicing errors.

A total of over 95,000 circRNAs have been identified in 12 plant species so far [15], including the model plant species *Arabidopsis thaliana* and *Oryza sativa* [5, 6]. Although the pervasiveness of circRNAs has been confirmed in plants, little is known about the circularization and the biological functions of plant circRNAs at present. Different from the biogenesis of circRNAs in animals, the flanking sequences of plant circRNAs do not seem to be enriched for repetitive elements or reverse complementary sequences [5, 6]. Recent studies in *A. thaliana* found that one circRNA generated from an exon regulates the expression of its parental gene by forming an R-loop [16], and a lariat-derived circRNA generated from an intron regulates gene expression and influences development [17].

As one of the most important edible oil seed crops in the world, peanut (*Arachis hypogaea* L.) is an autogamous allotetraploid legume (AABB, 2n = 40) with homoeologous A and B genomes that are derived from two diploids, *A. duranensis* (AA, 2n = 20) and *A. ipaensis* (BB, 2n = 20). To examine the scope of peanut circRNAs and to understand the features and possible functions in the regulation of peanut gene expression, we systematically investigated circRNAs in peanut using high-throughput RNA sequencing technology. Because seed size is an essential trait for crop breeders and is a major component of seed yield [18], all mRNAs were extracted from seeds of two representative peanut lines: ‘RIL 8106’ and ‘RIL 8107’, at 15 and 35 days after flowering (DAF). They were sequenced to analyze the roles of miRNAs in peanut seed expansion in our previous study [19]. Here, our study aimed to identify, validate and analyze the circRNAs expression profiles in peanut. The present study represents the first transcriptome-wide circRNAs identification in peanut, and the results not only prove the existence of circRNA, but also provide clues about the potential function of circRNAs in peanut.

## Methods

### Plant materials

Seeds of two peanut lines, ‘RIL 8106’ and ‘RIL 8107’, were bred and sown in the field at Henan Agricultural University, Zhengzhou, China. These two lines are eighth-generation recombinant inbred lines (RIL8) from a cross between two Virginia-type cultivars, and saved at Henan Agricultural University, Zhengzhou, China. The main difference between the two RILs is the pod size: ‘RIL 8106’ has medium-sized pods (3.2 cm long × 1.3 cm wide), and a 100-seed weight of 100 g, while ‘RIL 8107’ has super large pods (5.5 cm × 2.07 cm) with a corresponding 100-seed weight of 182 g. The samples were named C1 (‘RIL 8106’ at 15 DAF), C2 (‘RIL 8107’ at 15 DAF), T1 (‘RIL 8106’ at 35 DAF) and T2 (‘RIL 8107’ at 35 DAF). Seeds of each sample were collected from three independent plants at the same stage, and were pooled together for RNA isolation with three biological replicates.

### RNA extraction, cDNA library construction, and RNA sequencing

Total RNA was extracted from peanut seeds using Trizol reagent (Invitrogen, CA, USA) following the manufacturer’s protocol. The integrity and quality of the total RNA was evaluated using an Agilent 2100 Bioanalyzer (Agilent Technologies) and an RNA 6000 Nano Lab Chip Kit (Agilent Technologies, Boeblingen, Germany), with RIN (RNA integrity number) > 7.0. RNA quantity was measured using the NanoDrop 2000 spectrophotometer (Thermo Scientific, Wilmington, USA).

As per the manufacture’s instruction (Epicentre Ribo-Zero Gold Kit, Illumina, San Diego, USA), approximately 10 μg total RNA was subject to rRNA depletion. The RNA fragments were then reverse-transcribed to create the final cDNA library using the mRNA-Seq sample preparation kit by following the recommended protocol (Illumina, San Diego, CA, USA). The prepared libraries were then sequenced on an Illumina Hiseq 4000 platform (LC Sceiences, Hangzhou, China), and 2 × 150 bp paired-end reads (PE150) were generated according to the standard Illumina protocol.

### Read mapping and transcriptome assembly

Prior to assembling, the low quality reads (reads containing sequencing adaptors; reads containing > 5% Ns; reads containing nucleotides with Q quality scores < 20) were removed. The clean paired-end RNA-seq reads (150 nucleotides) were first mapped to the two diploid *Arachis* reference genomes, *A. duranensis* and *A. ipaensis*, obtained from the peanut database (https://www.peanutbase.org/) using TopHat (v2.1.0). The mapped reads were assembled into known and novel linear transcripts using Cufflinks (v2.1.1). All transcripts were pooled and merged to generate the final transcriptome using Cuffmerge (v 2.1.1).

### Annotation of peanut circRNAs

All the unmapped reads were processed to build a database of fusion transcripts using TopHat-Fusion (v2.1.0), and then aligned with the transcripts from the assembled linear RNAs using the CIRCexplorer [20] and CIRI [21] to identify candidate circRNAs. The low-confidence back-spliced junction reads were filtered out by the computational pipeline. The transcripts predicted by the two algorithms with back-spliced junction reads ≥2 were identified as candidate circRNAs.

### Transcript abundance estimation and differential expression

The number of reads spanning the back-splicing junction was used to quantify the expression of circRNAs. Host genes producing individual circRNAs were identified by matching the genomic location of the circRNAs with the location of genes detected by TopHat/Cufflinks using BED tools. Transcripts were considered novel if they did not overlap with annotated mRNAs. The circRNA host genes were mapped to terms in the GO database to determine their functions.

The aligned read files were processed with Cufflinks (v2.1.1), which uses the normalized RNA-seq fragment counts to measure the relative abundance of individual transcripts. Cuffdiff (v2.1.1) was used to calculate the Fragment Per Kilobase of exon per Million fragments mapped (FPKM) values for the circRNAs and mRNAs in all samples. Differential expression analysis of circRNAs was conducted using a two-tailed Student’s t-test and the SPSS Statistics v19.0 software package (IBM, NY, USA). The false discovery rate (FDR) was calculated to correct the *P* value, and its threshold was set at < 0.05. The circRNAs were considered to be significantly differentially expressed only when |log_2_ fold-change| ≥ 1 and *P* value ≤0.05.

### Validation of circRNAs

To validate the circRNAs identified in total RNA extracted from peanuts, qRT-PCR assays were carried out with divergent primers designed using Primer 5. For qRT-PCR, first-strand cDNA was synthesized from 1 μg total RNA with random hexamer primers using the First Strand cDNA Synthesis kit (Takara, Dalian, China). qRT-PCR conditions were as follows: an initial 3 min denaturation at 98 °C followed by 35 cycles of 45 s at 98 °C, 35 s at the appropriate annealing temperature (depending on the divergent primer set used), and 30 s extension at 72 °C, with a final extension at 72 °C for 10 min. The *ELF1B* gene was used as the internal control for normalization of gene expression [22]. RNase R treatment was conducted according to the reference [23] to confirm the circular form. PCR products were also separated by agarose gel electrophoresis and the expected bands were individually excised, purified, and directly sequenced using the Sanger method.

## Results

### Identification of circRNAs in peanut

To obtain sufficient transcriptome data, we separately deep-sequenced RNA samples extracted from seeds at 15 DAF and 35 DAF from two isogenic lines (‘RIL 8106’ and ‘RIL 8107’) with three biological replicates (Additional file 1: Table S1). After removing the low quality reads, a total of 1.353 billon paired-end reads (150 nucleotides in length) were generated (Additional file 1: Table S2). The sequencing reads were then mapped to the two diploid *Arachis* reference genomes (*A. duranensis* and *A. ipaensis*) to screen for back-spliced reads, which contain back-splicing junctions formed by the joining of a splice donor site to an upstream splice accepter. In total, 26,460,204 candidate back-spliced junction reads were identified in the four samples.

**Fig. 1:**
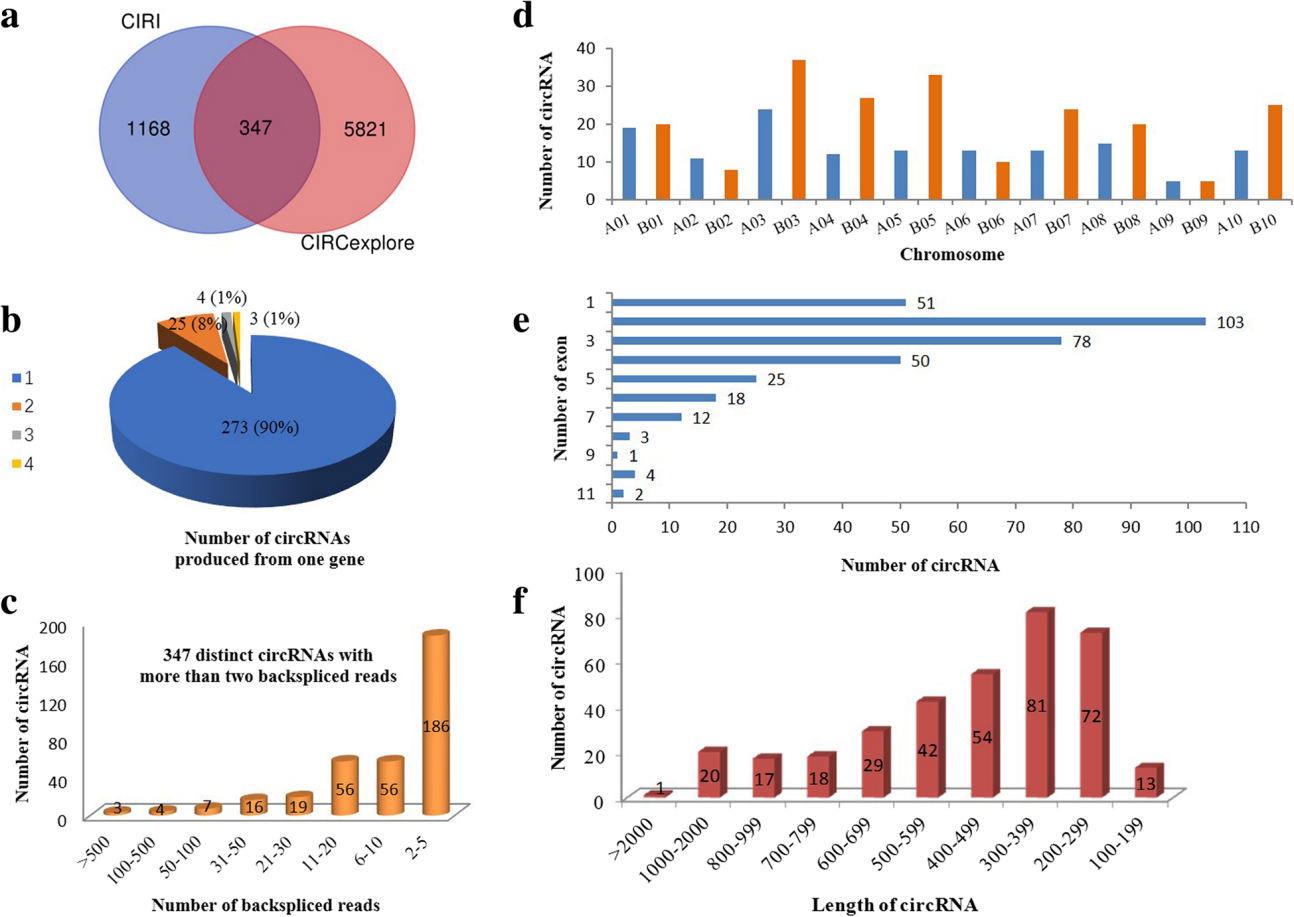
Identification and characteristics of circRNAs in peanut. **a** Genomic origin of circRNAs described in this study. **b** Number of circRNAs produced from the corresponding parent gene (347 circRNAs from 305 parent genes). **c** The number of circRNAs and back-spliced reads in four samples. **d** Distribution of circRNAs on 20 chromosomes. **e** The number of exons in each circRNA. **f** The length distribution of circRNAs

Circular RNA candidates were predicted by CIRCexplorer [20] and CIRI [21] computational pipelines. A total of 6168 distinct circular RNA candidates were identified by CIRCexplorer; of these, 1082 circular RNAs (17.5%) were generated from introns (intronic circRNA), and the remaining 5086 circular RNAs (82.5%) were generated from exons of a single protein-coding gene (exonic circRNA,). For the CIRI, a total of 1515 circular RNAs were identified including 61 intronic circRNAs (4.0%) and 1454 (96.0%) exonic circRNAs. Considering the possible false positive candidates, 347 exonic circRNAs were predicated by the two methods were used for the following analysis (Fig. 1a).

The number of circRNAs produced from their corresponding linear mRNAs showed that some parent genes produced more than one circRNA (347 circRNAs from 305 host genes), although most genes (90%, 273 out of 305) produced only one circRNA (Fig. 1b, Additional file 1: Table S3). There were three genes that each produced four circRNAs, and four genes which each generated three circRNAs, and the remaining 25 genes, which each generated two circRNAs. Our analysis showed that all circRNAs contained at least two unique back-spliced reads (Fig. 1c), and the average reads number was 24. circRNA604 contained the most back-spliced sites (1913) in all the samples. Genomic mapping revealed that these circRNAs are distributed widely and unevenly on the 20 peanut chromosomes (Fig. 1d, Additional file 1: Table S4). For example, 37 circRNAs were generated from genes on chromosome B03 when compared with five circRNAs produced from genes both on A09 and B09. We also calculated the exon numbers for all circRNAs (Fig. 1e). Only 51 circRNAs were derived from a single exon, and the maximum number of exons in a circRNA was predicted to be 11. The largest group consisted of circRNAs that contained two exons, accounting for 30% (103 out of 347), followed by a group containing three exons (23%, 78 out of 347). The average length of the circRNAs was 487 bp, and the longest and shortest were 2783 bp and 145 bp, respectively (Fig. 1f). These results indicate that circRNAs may represent one of the largest RNA families in the peanut transcriptome, similar to the two model plants *O. sativa* [5] and *A. thaliana* [6].

### Expression profiles of circRNAs in peanut

**Fig. 2:**
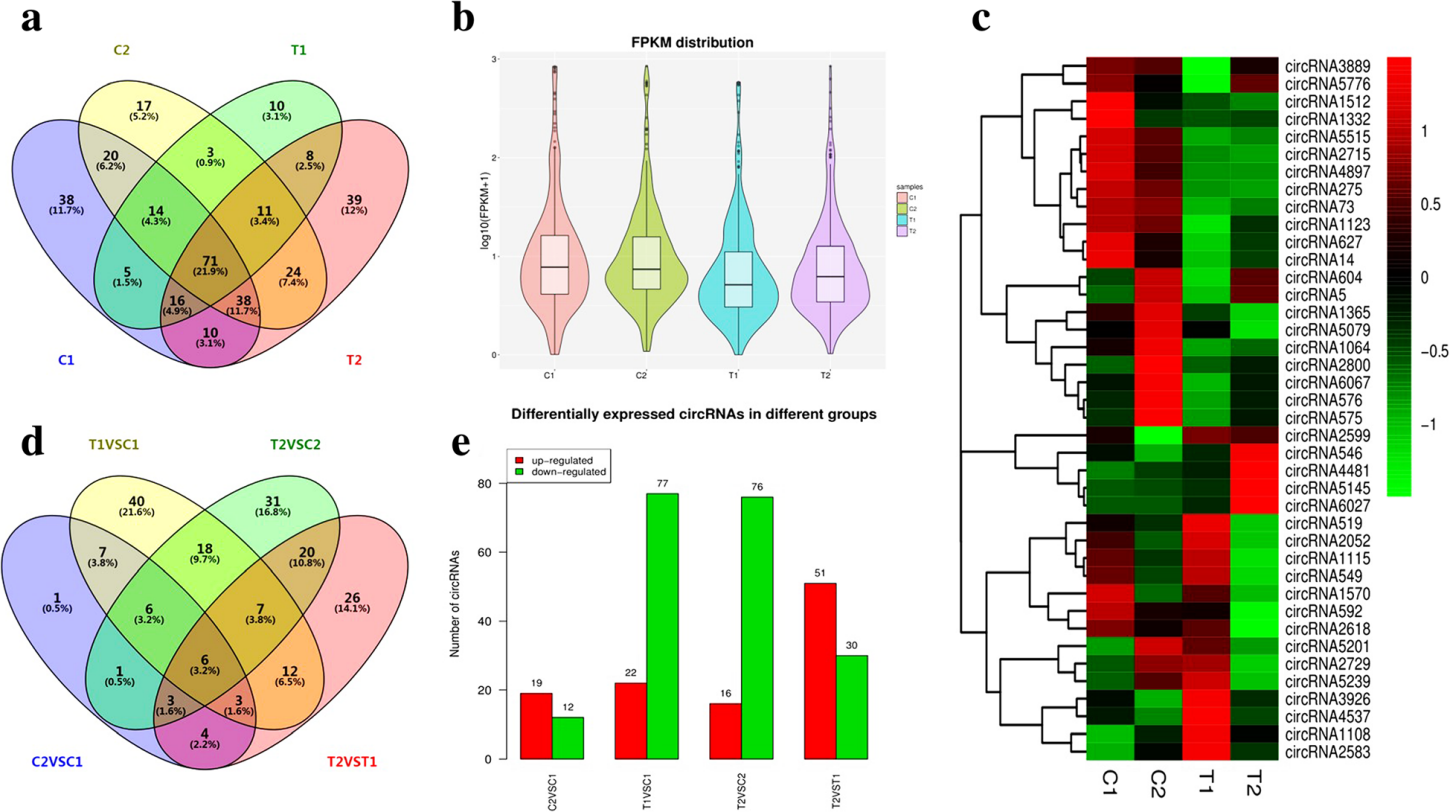
Expression profiles of circRNAs in peanut. **a** Venn chart of circRNAs detected in each sample. **b** Violin plot of relative abundance of circRNAs in four samples. **c** Clustered heat map of 40 differentially expressed circRNAs. **d** Venn chart of differentially expressed circRNAs. **e** Regulation of differentially expressed circRNAs in different comparisons. Note: C1 (‘RIL 8106’ at 15 DAF), C2 (‘RIL 8107’ at 15 DAF), T1 (‘RIL 8106’ at 35 DAF) and T2 (‘RIL 8107’ at 35 DAF)

All of the circRNAs identified in the four samples were analyzed together (Fig. 2a). The numbers of circRNAs detected specifically in the four lines were not equally compared, including 38 in C1, and 17 in C2, 10 in T1 and 39 in T2. There were 71 circRNAs (20.7%) were common to all four samples.

We normalized the expression profiles of the circRNAs based on their FPKM values, which permits quantitative comparisons of the levels of each circRNA between the different samples. The relative expression of circRNAs at 35 DAF was down-regulated compared with 15 DAF (Fig. 2b). Cluster analysis of 40 circRNAs randomly selected from the top 100 highly expressed circRNAs (more junction reads) revealed that diverse expression patterns of circRNAs not only in comparisons between the two developmental stages (15 and 35 DAF), but also in comparisons of the two different peanut lines at the same developmental stage (Fig. 2c).

We next analyzed the differentially-expressed circRNAs in the different group comparisons (|log_2_ fold-change| ≥ 1 and *P* value ≤0.05). We detected 26, 40, 36, and one specific circRNAs that were differentially expressed in the T2 vs. T1, T1 vs. C1, T2 vs. C2, and C2 vs. C1 comparisons, respectively, and six circRNAs that were differentially expressed in all four groups (Fig. 2d). Among the circRNAs that were differentially expressed in comparisons of 15 DAF and 35 DAF seeds in the same line, we found that more circRNAs were down-regulated than up-regulated (77 down-regulated and 22 up-regulated in T1 vs. C1, and 76 down-regulated and 16 up-regulated in T2 vs. C2) (Fig. 2e). However, more circRNAs were up-regulated than down-regulated (19 and 12 in C2 vs. C1, and 51 and 30 in T2 vs. T1, respectively) in the large-seeded line ‘RIL 8107’ compared with the medium-seeded line ‘RIL 8106’ at the same developmental stage. These results suggest that the differentially expressed circRNAs might play a dominant role in regulating seed development.

### Structural formation of circRNAs in peanut

Based on the annotated transcripts, gene structure of the circRNAs derived from the same linear mRNA can be inferred by examining the back-splicing sites to determine the circularization position.

**Fig. 3:**
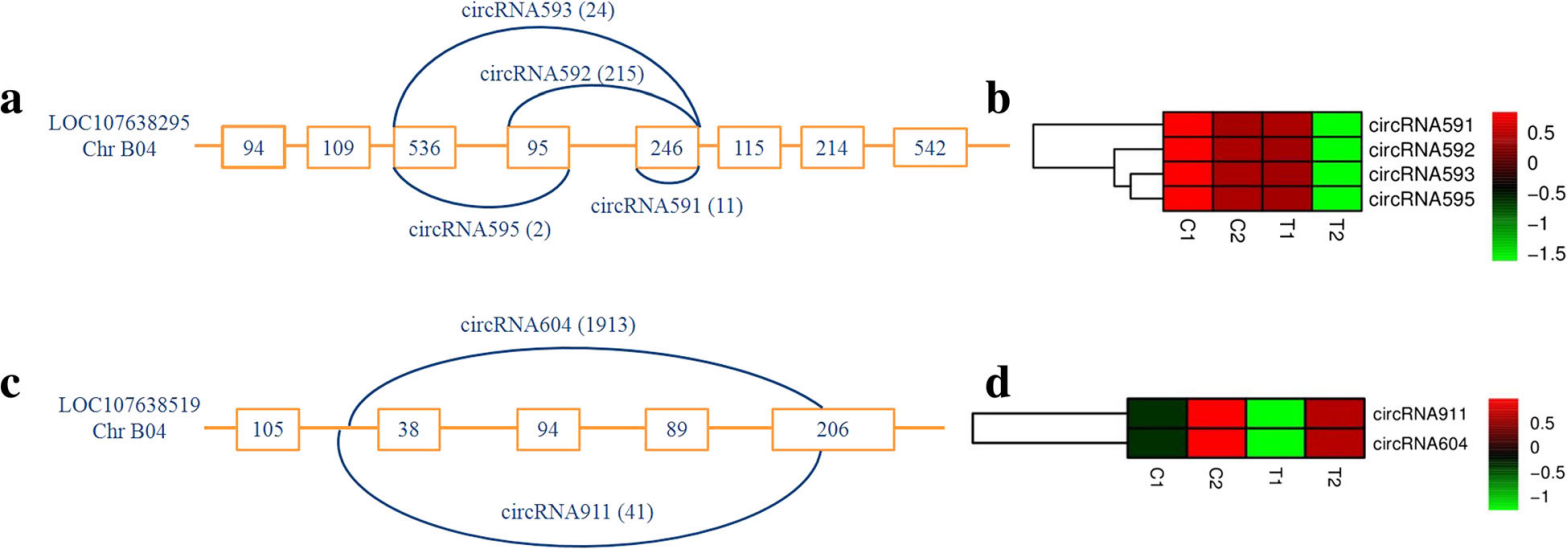
Visualization of circRNAs generated from the same parent gene in peanut. **a** Four exonic circRNAs generated by alternative circularization. **b** Clustered heat map of four circRNAs generated from the same parent gene LOC107638295. **c** Two circRNAs generated from the same parent gene LOC107638519. **d** Clustered heat map of two circRNAs generated from the same parent gene LOC107638519. Square frame means the exons, and number in square frame means the length of exon. The supported unique reads were presented in the parentheses followed the circRNA. Note: C1 (‘RIL 8106’ at 15 DAF), C2 (‘RIL 8107’ at 15 DAF), T1 (‘RIL 8106’ at 35 DAF) and T2 (‘RIL 8107’ at 35 DAF)

One gene, LOC107638295, which consists of eight exons and encodes a subtilisin-like serine protease, was found to yield four circRNAs (Fig. 3a). The number of exons in these circRNAs varied from one to three, and the lengths were between 246 bp and 876 bp. The corresponding exon boundaries of these circRNAs were not completely following the AG-GT rules. Alternative back-splicing circularization pattern (same parental gene, different back-splices) was identified. For example, circRNA591 shared the same acceptor sites with both circRNA593 and circRNA595, while also shared the same donor sites with circRNA592. These circRNAs also showed consistent expression patterns among the four samples (Fig. 3b).

circRNA604, a predominant circRNA isoform generated from LOC107638519, was found to have the largest number of back-spliced reads. Bioinformatic analysis indicated that LOC107638519 contains 12 exons and produced two circRNAs, circRNA604 and circRNA911 (Fig. 3c). The genomic structure showed that circRNA604 (384 bp) consisted of a small fragment of one intron (70 bp), three complete exons (38 bp, 94 bp and 89 bp, respectively) and 93 bp of the last exon, with a full-length of 206 bp. Another isoform, circRNA911 (393 bp), had almost the same sequence as circRNA604 except for an extra 9 bp of the same intron, and it was similar to circRNA604 in that expression level and expression pattern (Fig. 3d). The counterpart gene on A04, LOC107482719, also generated two isoforms (circRNA5 and circRNA3592) with the same length and position, similar to those from B04.

### Differentially-expressed circRNAs are related to seed development in peanut

In order to shed light on the potential function of circRNAs in seed development, we performed GO enrichment analyses of the host genes that produced differentially expressed circRNAs. We found that these genes are involved in a broad range of molecular functions, such as “nutrient reservoir activity” and “DNA binding”, as well as various biological progresses such as “cell division” and “mucilage metabolic process involved seed coat development” and “syncytium formation” (Additional file 2: Figure S1).

To further uncover the possible regulatory functions of circRNAs, we focused on the top GO terms involved in seed development, and then screened out the circRNAs that were differentially-expressed between 15 DAF and 35 DAF in the same line (T1 vs. C1, and T2 vs. C2). GO enrichment analysis of the T1 vs. C1 comparison revealed that the host genes were significantly enriched (*P* < 0.05) in “cell division” and “nutrient reservoir activity”. All the circRNAs related to nutrient reservoir activity were significantly up-regulated, and all circRNAs involved in cell division were significantly down-regulated. GO terms in the T2 vs. C2 comparison showed that the host genes were significantly enriched (*P* < 0.05) in “mucilage metabolic process involved seed coat development”, besides of “cell division”. Most of the circRNAs related to these GO enrichment were significantly down-regulated, which indicates that these circRNAs are involved in seed development between 15 and 35 DAF in peanut.

**Fig. 4:**
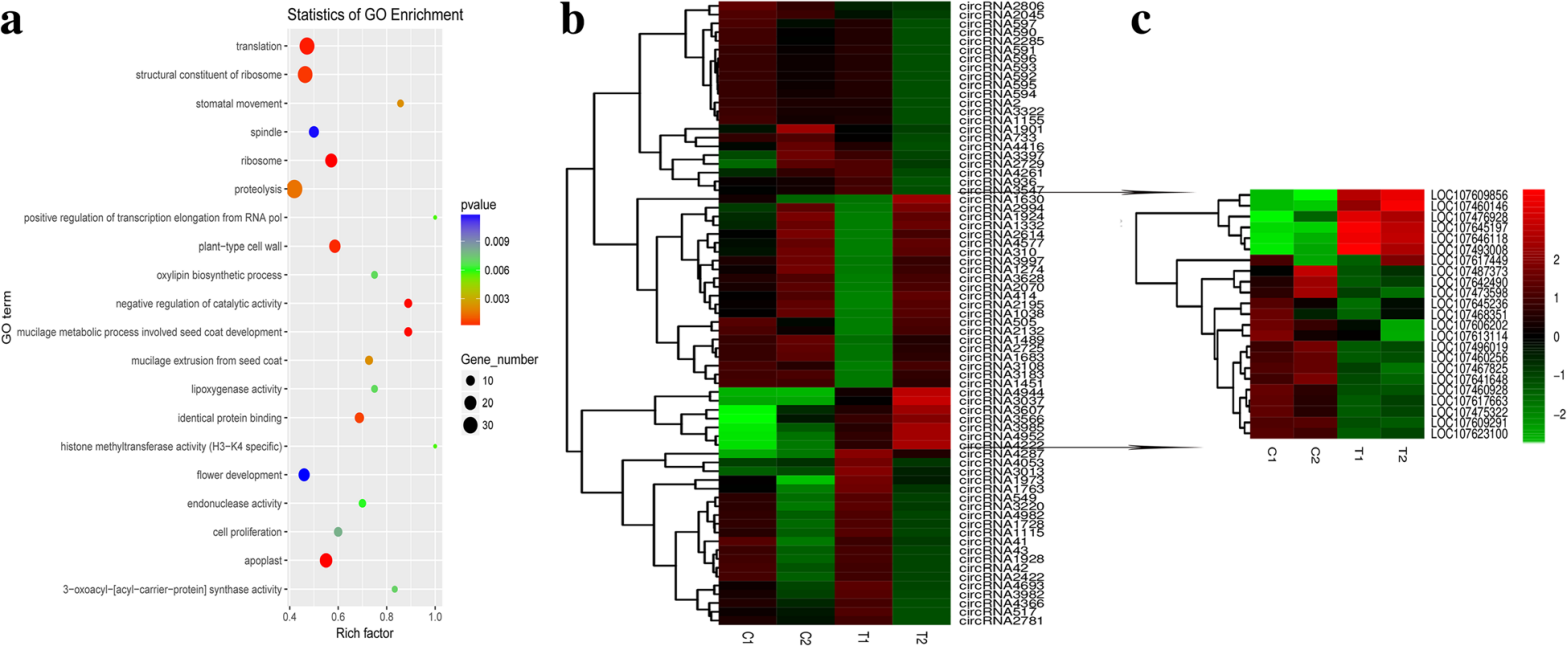
GO enrichment analysis of the parent genes generating differentially expressed circRNAs in T2 vs. T1 comparison. **a** GO enrichment scatter plot of T2 vs. T1 comparison. **b** Clustered heat map of 71 differentially expressed circRNAs corresponding to enriched parent genes. **c** Clustered heat map of 23 parent genes generating 29 circRNAs up-regulated in the T2 vs. T1 comparison. Note: C1 (‘RIL 8106’ at 15 DAF), C2 (‘RIL 8107’ at 15 DAF), T1 (‘RIL 8106’ at 35 DAF) and T2 (‘RIL 8107’ at 35 DAF)

We then used GO enrichment analysis to investigate whether circRNAs might regulate seed size in the two peanut lines at 15 and 35 DAF (C2 vs. C1, and T2 vs. T1). The top 20 most significant GO terms in the T2 vs. T1 comparison are shown in Fig. 4a. We found that GO terms related to seed development, including nutrient reservoir activity, mucilage metabolic processes involved in seed coat development, and cell proliferation, were all significantly enriched (*P* < 0.05). A clustered heatmap of the set of enriched circRNAs revealed that those classified in the same GO terms showed similar expression patterns (Fig. 4b). We found 29 circRNAs that were up-regulated in the T2 vs. T1 comparison, and the expression of their 23 corresponding host genes (for example, circRNA1630 and its host gene LOC107617449) showed similar changes in expression.

### Validation of peanut circRNAs

**Fig. 5:**
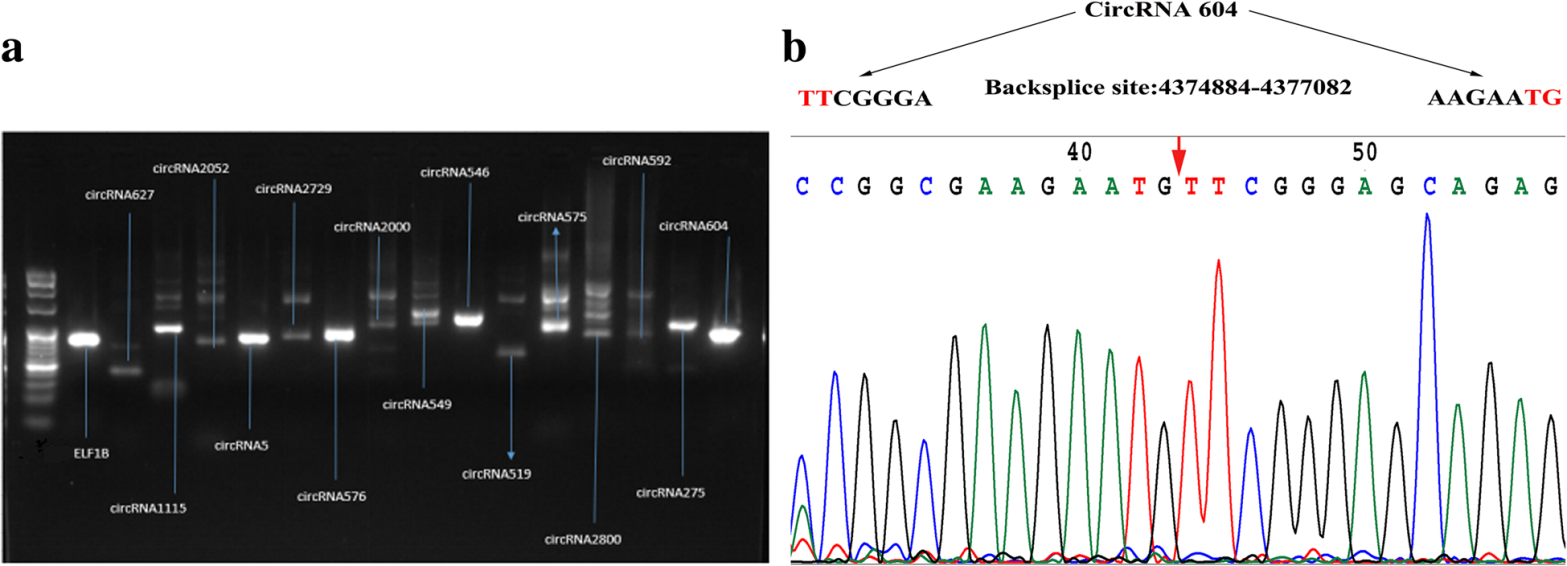
Validation of circRNAs by qRT-PCR and Sanger sequencing. **a** The expected fragments amplified with divergent primers (covering the back-splicing junction) in 15 circRNAs. **b** circRNA604 was further confirmed by Sanger sequencing. The red arrow represents the back-splicing site of circRNA604

To confirm our identification of circRNAs, 15 highly-expressed circRNA candidates were selected for experimental validation using quantitative reverse transcription PCR (qRT-PCR) and digestion with RNase R exonuclease. A set of divergent primers (Additional file 1: Table S5) was designed for qRT-PCR with *ELF1B* as the internal gene expression control. The PCR amplified DNA fragments corresponding to 15 circRNAs are shown in Fig. 5a. A single discreet product of the predicted size was specifically amplified from six of the 15 circRNAs. The remaining circRNAs also gave other non-specific bands in addition to the predicted DNA fragments, which could result from multiple rounds of reverse transcription around a circular RNA template [24]. The back-splicing junctions present in the six circRNAs were further validated by Sanger DNA sequencing (Fig. 5b). Resistance to digestion with RNase R exonuclease further confirmed that this RNA species was circular in form (Additional file 4: Figure S3). The results of this experiment indicate that circRNAs identified in the RNA-seq datasets are reliable.

## Discussion

Circular RNAs are a newly validated class of endogenous non-coding RNAs that show resistance to exonucleases such as RNase R compared with their linear counterparts [25]. Due to rapid advances in the development of combined high-throughput sequencing and bioinformatics analysis tools, circRNAs were recently discovered and have been identified and characterized in the transcriptomes of a variety of eukaryotic organisms [2, 3, 5, 8], but not in peanut until now. In this study, we identified 347 circRNAs in the transcriptome of peanut seeds using two algorithms. Compared with published results from human [4, 26], a much smaller number of circRNAs were identified in peanut, which likely due to temporal and spatial specific expression of circular RNAs [3]. The number of circRNA candidates identified in 12 plant species varied greatly, such as 5323 circular RNAs in *Glycine max* [27] and 39 circRNAs in *Hordeum vulgare* [28]. Even in the same species, the number of circRNAs identified by different research groups showed obvious difference, for example, Ye et al. [6] reported 889 circular RNA candidates while Dou et al. [29] identified only 13 circular RNA candidates in *Arabidopsis*. The possible reasons involved in the sequencing quality, studied tissues, specifically designed RNA-seq libraries and different algorithms used, etc.

circRNAs were previously thought to be by-products of pre-mRNA splicing due to their low abundance [8, 30]. Recent studies have shown that certain circRNAs are predominately expressed from a single genomic locus and are present at substantial levels, suggesting that circRNAs are a naturally occurring RNA family with regulatory potential [3, 31]. Recently, circRNAs were identified in tomato and involved in fruit pigment accumulation [32]. We also characterized the expression profiles of circRNAs and speculated their possible functions in regulating gene expression. Consistent with previous studies, we found that numerous abundant circRNAs were specifically expressed at two developmental stages and were differentially expressed between two lines in this study, which suggests that they might have specific functions in seed development in peanut.

In mammals, “direct back-splicing” has been proposed to be a common mechanism for exonic circRNAs formation [2, 8, 31]. Exon circularization were found to depend on flanking intronic complementary sequences, and competition between inverted repeated ALU pairs leads to alternative circularization were further studied in human [20]. All the circRNAs identified in our study were predicted to be generated from exons of annotated coding genes, and the location of back-splicing sites was defined based on the mapping reads. Although most host genes produced only one circRNA, previous studies have also shown that a single parental gene can produce multiple circRNAs [23, 33]. Similar to the splicing patterns in rice [5], multiple circRNAs were generated by alternative back-splicing from exon circularization of the same parental gene. But putative intronic complementary sequences were only detected in 20 out of 2354 circRNAs in rice. Neither complementary sequences nor repeat sequences were found in exon circularization of RNAs in *A. thaliana* [29]. These studies suggest that there is other mechanisms may be involved in plant circRNAs generation, but due to the scarcity of plant circRNAs, the possibility that some plant circular RNAs contain complementary sequences in their flanking regions, can’t be ruled out.

Various bioinformatics prediction tools, such as CIRCexplorer [20], CIRI [21], circRNA_finder [34], KNIFE [35], find_circ [3] and Segemehl [36], were developed to identify circRNAs in various species. They showed pros and cons in the precision and sensitivity in circRNA prediction. Two algorithms were used in this study to reduce false positive circRNAs as much as possible. Interestingly, though 1082 and 61 ciRNAs were predicted by CIRCexplorer and CIRI, respectively, no interaction was found. circRNAs were classified into ten types based on the annotated genomic features, and the results showed that most plant circRNAs were generated from annotated genes including both exonic and intronic regions [15]. In view of the intronic circRNAs was predicted to be widespread in other plants, and was proved to regulate gene expression and influence development of *Arabidopsis* recently [17], circular RNAs generated from intron was not be predicted here, and more tools should be used in peanut in the near future.

Complexity of circRNA expression was found in many studies and has been recently reviewed [37]. The expression patterns of circRNAs derived from the same linear mRNA were diverse between the two peanut lines and in comparisons of two developmental stages. Some circRNAs were more abundantly expressed than were their parental linear mRNA transcripts, and their expression was even independent of the related linear isoforms. One abundant circRNA, circHIPK3, which is derived from exon 2 of the HIPK3 gene, had a higher expression level than did the HIPK3 mRNA. This circRNA, but not HIPK3 mRNA, was also shown to significantly affect cell proliferation in human cells [23]. Most circRNAs showed inconsistencies between the number of back-spliced reads and expression profiles (FPKM values). All of these results suggested the complicated expression of circRNAs in peanut.

Although the functions of most circRNAs remain largely unexplored, they are known to be involved in the sequestration of miRNAs or proteins, modulation of transcription and interference with mRNA splicing, and even in translation to produce polypeptides in animals [3, 23]. In contrast, the circRNAs exploration in plants is inadequate and limited in several plants such as *Arabidopsis* [6, 29], rice [5], maize [38] and tomato [39]. We also predicted the potential “sponges” function of these circRNAs identified in our study, and the results showed that a single circRNA was predicted to contain at most two predicted binding sites for one miRNA (Additional file 3: Figure S2, unpublished data). Therefore, we propose that it is more common for circRNAs working as miRNA “sponges”, but lack of adequate evidence in peanut so far. Even though several abundant circRNAs that act as miRNA “sponges” have been confirmed to regulate miRNA expression, like CDR1as to miR-7 [3], circHIPK3 to miR-124 [23] and circLMO7 to miR-378a-3p [40], there are recent publications that suggest that circRNAs do not necessarily function as miRNA “sponges” in mammalian cells [26]. So the role of circRNAs and their associated molecules in gene regulation, such as miRNAs or proteins, needs to be established by further experimental identification and characterization in peanut.

## Conclusions

Our study provides the first study of circRNAs generated from two peanut lines with different seed sizes at two developmental stages. We characterized the genomic structures and expression profiles of circRNAs to gain insight into the features of circRNAs. The results revealed that circRNAs are widespread and differentially expressed between lines during seed development in peanut.

## Additional files

Additional file 1: **Table S1.** Raw data of rRNA-depleted sequencing in peanut. **Table S2.** A detailed summary of RibominusSeq data after primary quality control for each sample. **Table S3.** Number of circRNAs derived from the same hosting gene. **Table S4.** Distribution of 347 circRNAs on 20 chromosomes of peanut. **Table S5.** Sequence of divergent primer pairs designed for 15 circRNAs. (XLS 44 kb)

Additional file 2: **Figure S1.** GO enrichment analyses of the host genes generating differentially expressed circRNAs. (a), (b) and (c) represent GO enrichment scatter plots of C2 vs. C1 comparison, T1 vs. C1 comparison and T2 vs. C2 comparison, respectively. (TIF 2404 kb)

Additional file 3: **Figure S2.** The interaction network between circRNAs and miRNAs. A magnified network showed 33 circRNAs (Green) and miRNAs (Purple). The host genes (Orange) which generated circRNAs were also shown. (TIF 970 kb)

Additional file 4: **Figure S3.** qRT-PCR for the abundance of circRNA604 treated with RNase R. ELF1B was used as control. The amount of circRNA604 was normalized to the value measured in the mock treatment. (TIF 58 kb)

## Data Availability

All data supporting the findings in this study are included within the manuscript and the supplementary files. The two peanut lines analyzed in this article, ‘RIL 8106’ and ‘RIL 8107’, are saved at Henan Agricultural University (Zhengzhou, China), and are available from the corresponding author on reasonable request.

## Abbreviations

ABA: Abscisic acid
circRNA: Circular RNA
DAF: Days After Flowering
FPKM: Fragment Per Kilobase of exon per Million fragments mapped
GO: Gene Ontology
lncRNA: Long non-coding RNA
qRT-PCR: Quantitative Real-time PCR
TGS: Third-Generation DNA Sequencing
