# Supplementary figures and images for: Genome-wide identification of circular RNAs in peanut (Arachis hypogaea L.)
Source: BMC Genomics. 2019 Aug 15;20:653. doi: 10.1186/s12864-019-6020-7 (PMC6694679; doi:10.1186/s12864-019-6020-7)

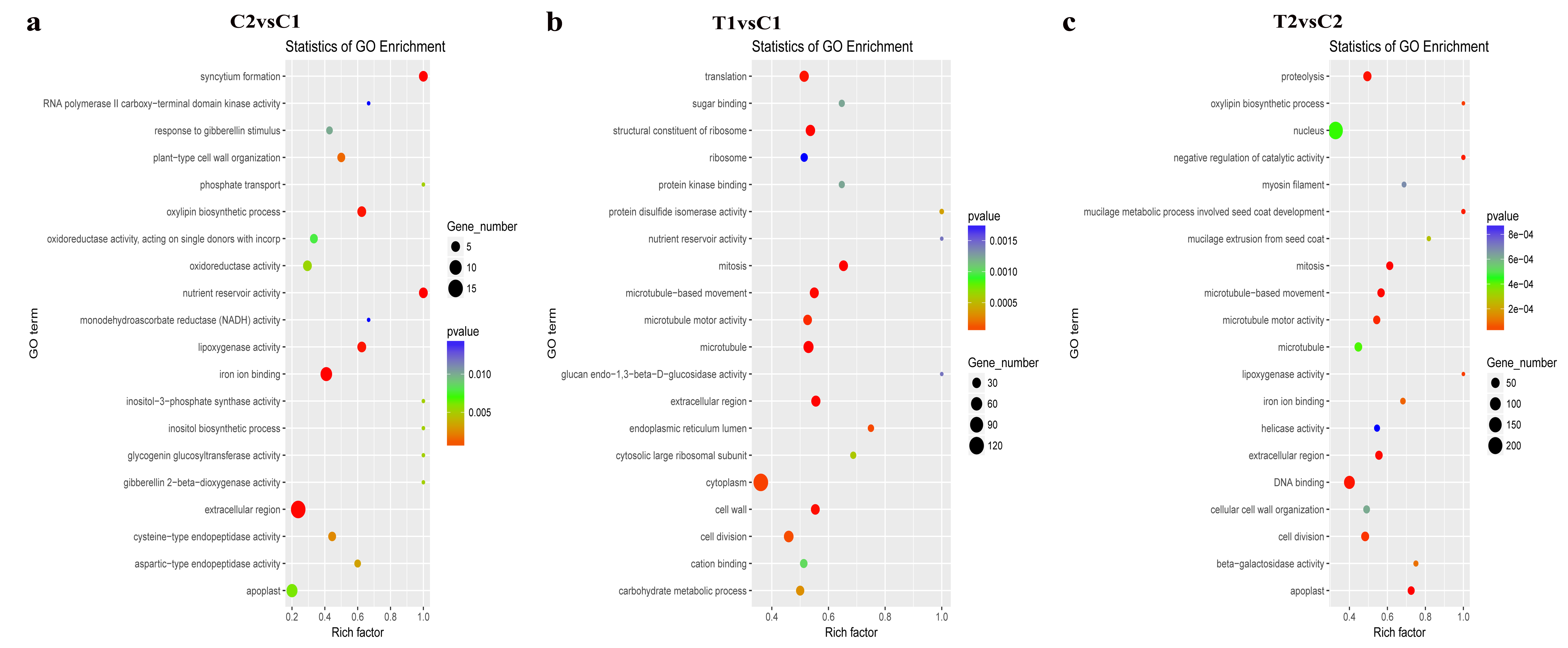

Supplement: Supplementary file 2 — Figure S1. GO enrichment analyses of the host genes generating differentially expressed circRNAs. (a), (b) and (c) represent GO enrichment scatter plots of C2 vs. C1 comparison, T1 vs. C1 comparison and T2 vs. C2 comparison, respectively. (TIF 2404 kb) [file 12864_2019_6020_MOESM2_ESM.tif]

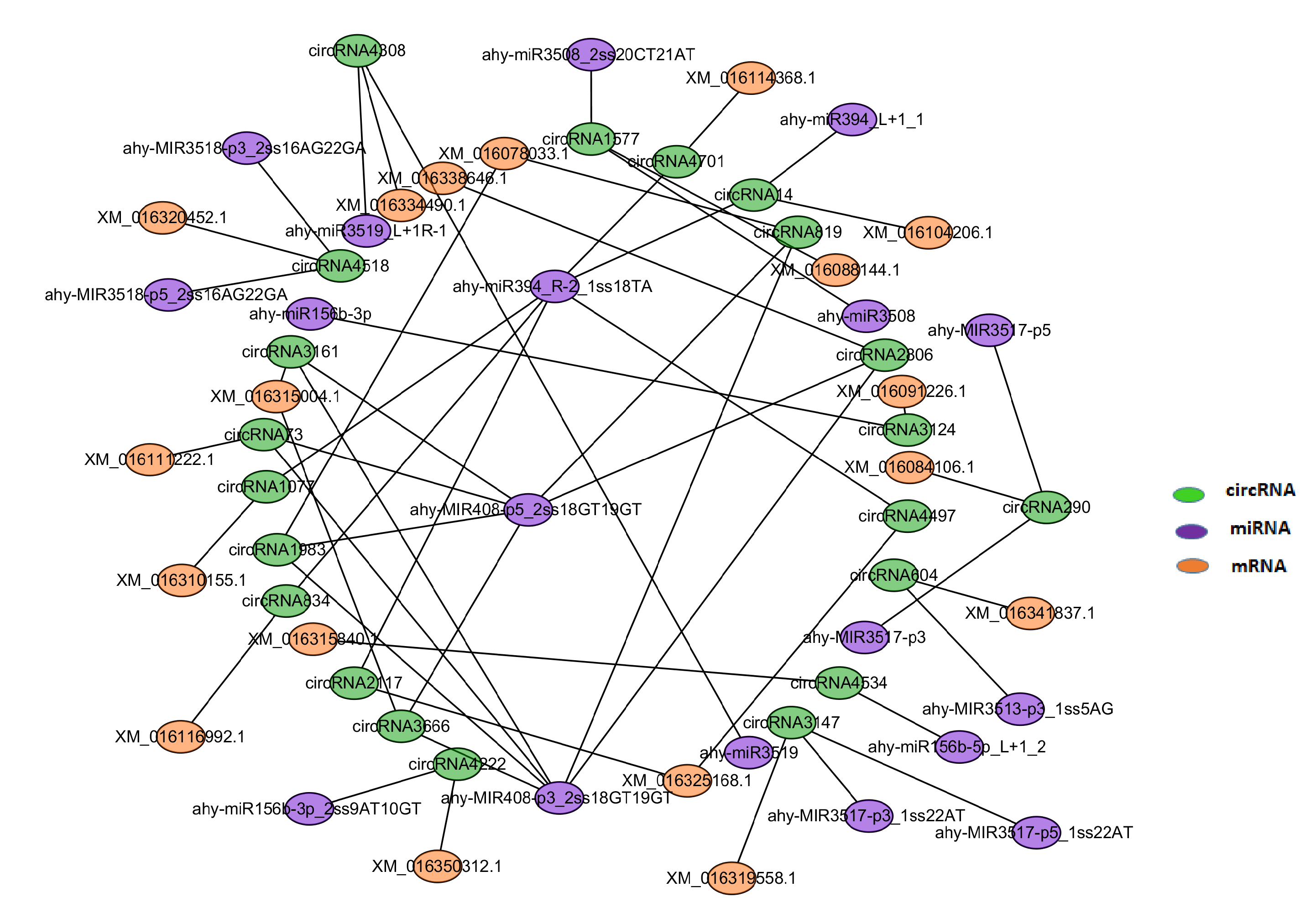

Supplement: Supplementary file 3 — Figure S2. The interaction network between circRNAs and miRNAs. A magnified network showed 33 circRNAs (Green) and miRNAs (Purple). The host genes (Orange) which generated circRNAs were also shown. (TIF 970 kb) [file 12864_2019_6020_MOESM3_ESM.tif]

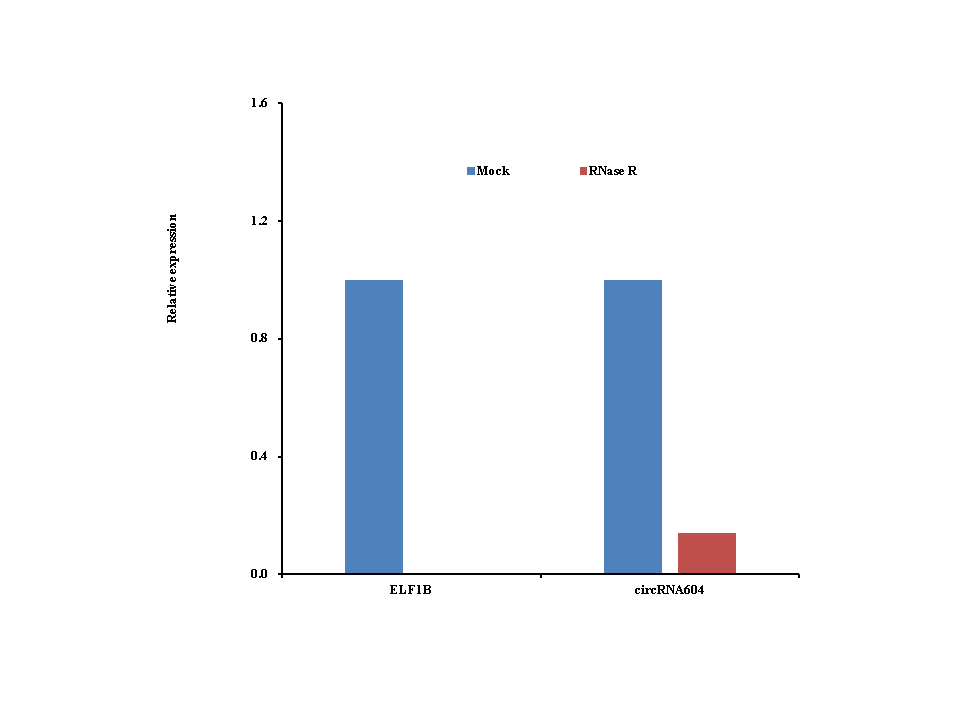

Supplement: Supplementary file 4 — Figure S3. qRT-PCR for the abundance of circRNA604 treated with RNase R. ELF1B was used as control. The amount of circRNA604 was normalized to the value measured in the mock treatment. (TIF 58 kb) [file 12864_2019_6020_MOESM4_ESM.tif]
